# Supplementary material for: Systematic benchmarking of high-throughput subcellular spatial transcriptomics platforms across human tumors
Source: Nat Commun. 2025 Oct 17;16:9232. doi: 10.1038/s41467-025-64292-3 (PMC12534522; doi:10.1038/s41467-025-64292-3)
Supplement: Supplementary file 1 — Supplementary Information [file 41467_2025_64292_MOESM1_ESM.pdf]

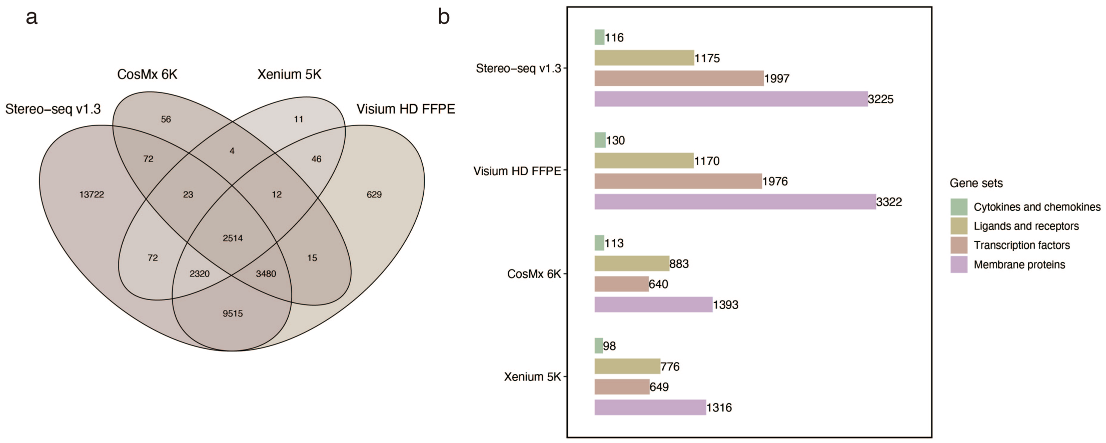

Supplementary Fig. 1 Comparison of genes detected by different ST platforms.
a. Venn diagram showing the overlap of detected genes across the four ST platforms.
b. Number of genes within selected functional categories detected by each platform, including cytokines and chemokines, ligands and receptors, transcription factors, and membrane proteins.

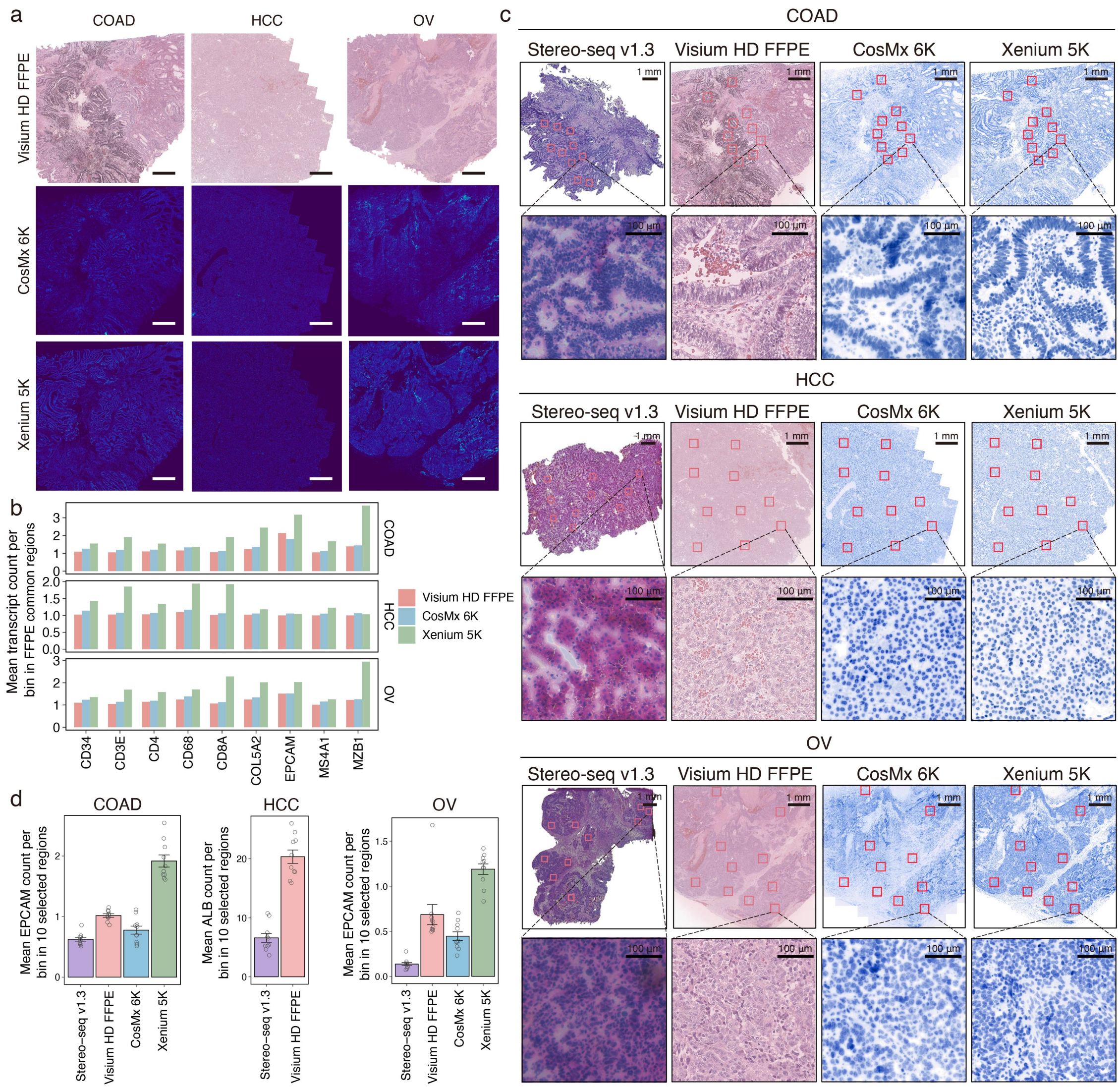

Supplementary Fig.2 Comparison of sensitivity for lineage marker genes.

a. Overlapping tissue regions for the three FFPE-based platforms: Visium HD FFPE, CosMx 6K, and Xenium 5K, shown for COAD, HCC, and OV samples (H&E staining for Visium HD FFPE; DAPI staining for CosMx 6K and Xenium 5K). Scale bars, 1 mm. **b.** Mean transcript counts per 8 × 8 µm bin for selected marker genes, computed across all bins with non-zero expression values over the shared FFPE regions shown in **a**. **c.** Spatial distribution of ten tumor regions with similar morphology (400 × 400 µm each), highlighted by red squares. Magnified views are shown in the bottom panels. **d.** Mean transcript count of marker genes (ALB for HCC; EPCAM for COAD and OV), averaged per bin per region across the ten selected tumor regions. Each hollow circle indicates the mean expression in one of the 10 regions (n = 10). Data are presented as mean values +/- SEM. Source data are provided as a Source Data file.

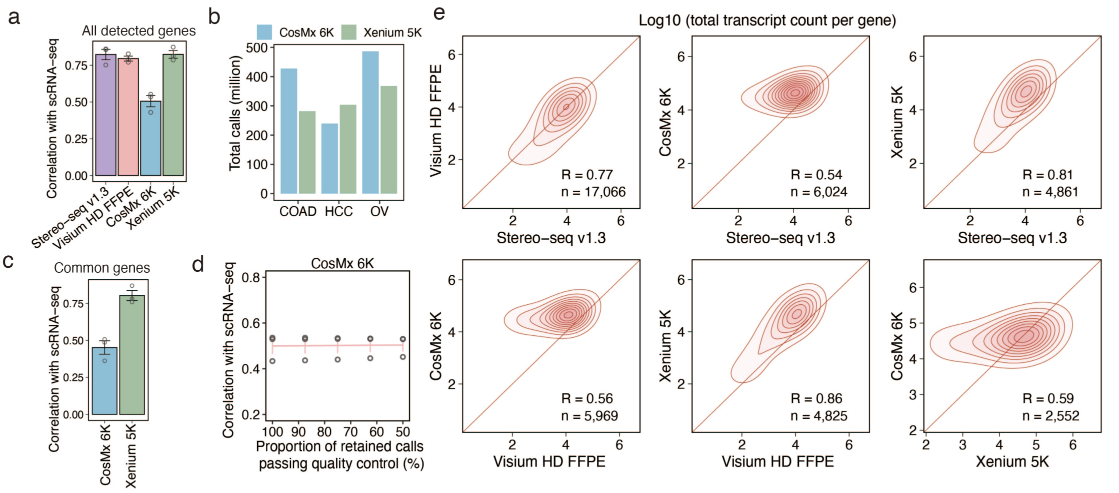

Supplementary Fig. 3 Correlation of gene expression levels across diverse platforms.

a. Gene expression correlations between each ST platform and matched scRNA-seq data, calculated using all detected genes. Pearson correlation coefficients are reported. Each hollow circle represents the correlation for one cancer type (n = 3). Data are presented as mean values +/- SEM. **b.** Total number of calls detected by CosMx 6K and Xenium 5K. **c.** Gene expression correlations between iST platforms and matched scRNA-seq data, calculated using 2,552 genes shared by CosMx 6K and Xenium 5K. Pearson correlation coefficients are reported. Each hollow circle represents the correlation for one cancer type (n = 3). Data are presented as mean values +/- SEM. **d.** Gene expression correlations between CosMx 6K and scRNA-seq after applying increasing quality control thresholds to CosMx 6K transcript calls. Pearson correlation coefficients are reported. Each hollow circle represents the correlation for one cancer type (n = 3). Data are presented as mean values +/- SEM. **e**. Pairwise gene expression correlations across ST platforms. For each gene, total transcript counts were averaged across the three cancer types and $\log_{10}$-transformed. The diagonal red line indicates a slope of 1, and color intensity corresponds to relative gene counts. R denotes the Pearson correlation coefficient, and n indicates the number of genes included in the analysis. Source data are provided as a Source Data file.

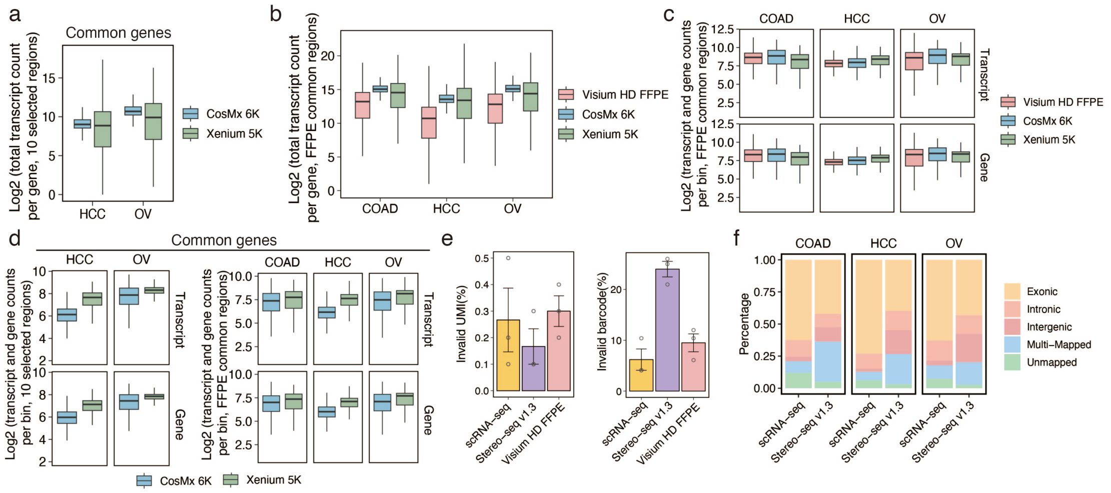

Supplementary Fig. 4 Comparison of sensitivity for entire gene panels.

a. Log_2-transformed total transcript counts for shared genes (n = 2,552) between CosMx 6K and Xenium 5K across the ten selected regions. Each data point represents one gene. Center lines indicate the median value, and lower and upper hinges represent the 25th and 75th percentiles, respectively. The whiskers denote 1.5× the interquartile range. **b.** Log_2-transformed total transcript count per gene across the shared regions of Visium HD FFPE, CosMx 6K, and Xenium 5K. Each data point represents one gene (n = 2,506 common genes). Center lines indicate the median value, and lower and upper hinges represent the 25th and 75th percentiles, respectively. The whiskers denote 1.5× the interquartile range. **c.** Log_2-transformed transcript and gene counts per 8 μm bin within the common regions of Visium HD FFPE, CosMx 6K, and Xenium 5K. All detected genes were included. Each data point represents one bin. Center lines indicate the median value, and lower and upper hinges represent the 25th and 75th percentiles, respectively. The whiskers denote 1.5× the interquartile range. **d.** Log_2-transformed transcript and gene counts per 8 μm bin within the ten selected regions (left) or shared FFPE regions (right). Analysis was based on the 2,552 genes shared between CosMx 6K and Xenium 5K. Each data point represents one bin. Center lines indicate the median value, and lower and upper hinges represent the 25th and 75th percentiles, respectively. The whiskers denote 1.5× the interquartile range. **e.** Percentage of reads failing UMI quality control (left) and reads with barcodes not matching the predefined barcode list (right). Hollow circles indicate percentage for different cancer types (n = 3). Data are presented as mean values +/- SEM. **f.** Proportion of reads mapped to exonic, intronic, and intergenic regions of the human genome, as well as reads that were multi-mapped or unmapped. Source data are provided as a Source Data file.

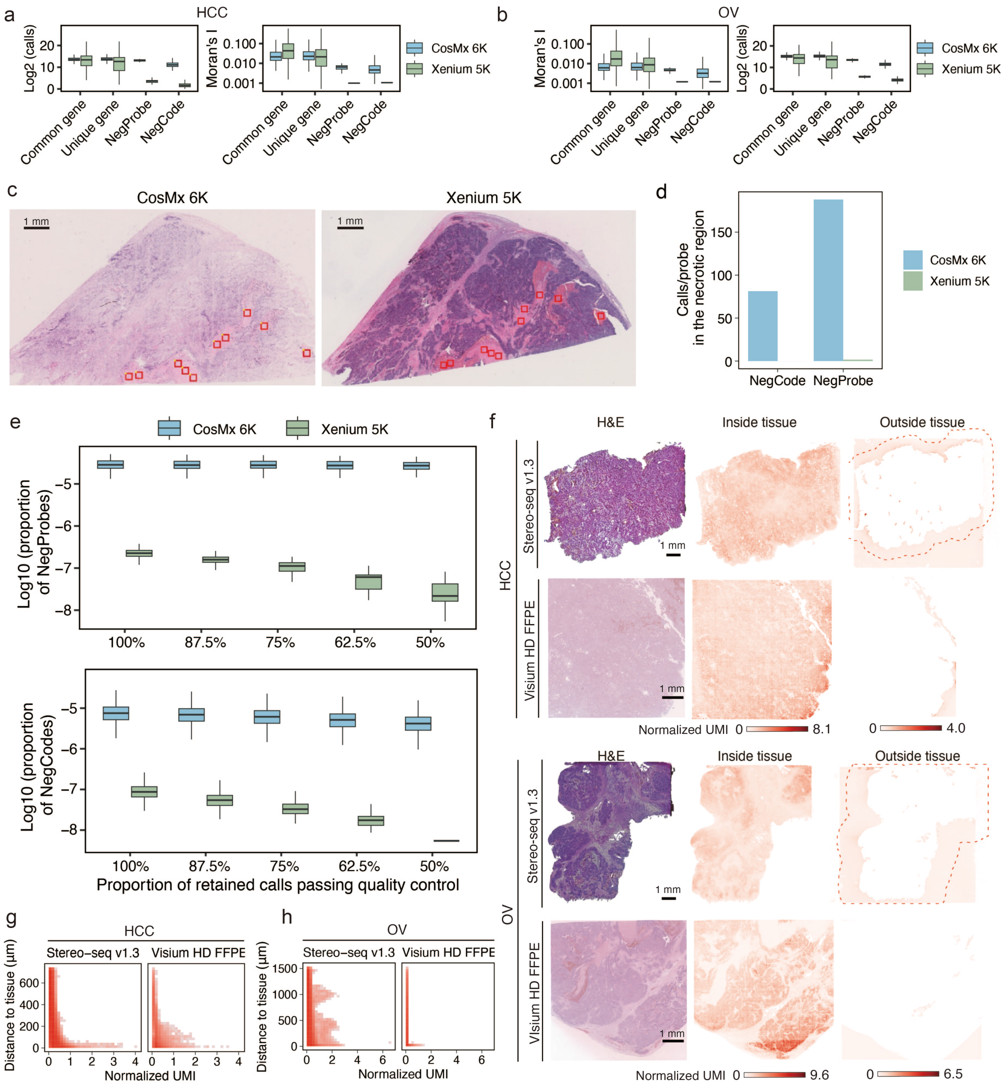

Supplementary Fig.5 Evaluation of negative control signals and transcript diffusion.

a-b. Total call counts and Moran's I for common genes (2,552), platform-specific genes (3,623 for CosMx 6K and 2,449 for Xenium 5K), negative probes (NegProbe, 20 for CosMx 6K and 40 for Xenium 5K), and negative codes (NegCode, 324 for CosMx 6K and 609 for Xenium 5K) detected by CosMx 6K and Xenium 5K across the shared regions in HCC (**a**) and OV (**b**). Each data point represents one target. Center lines indicate the median value, and lower and upper hinges represent the 25th and 75th percentiles, respectively. The whiskers denote 1.5× the interquartile range. **c**. H&E-staining of CosMx 6K and Xenium 5K OV samples. Red boxes indicate ten 250 × 250 μm regions manually selected from necrotic areas. **d**. Bar plot showing the average number of calls per negative control detected in the selected necrotic regions. **e.** Proportion of negative control signals in CosMx 6K and Xenium 5K datasets after applying increasing quality control thresholds to transcript calls. Center lines indicate the median value, and lower and upper hinges represent the 25th and 75th percentiles, respectively. The whiskers denote 1.5× the interquartile range. **f.** H&E staining and transcript distribution inside and outside the HCC and OV tissue regions. Red dashed

lines outline the Stereo-seq v1.3 regions used for diffusion analysis. Color intensity indicates mean-normalized transcript count of each 8 × 8 μm bin. **g-h.** Diffusion evaluation in HCC (**g**) and OV (**h**). The x-axis and y-axis represent the mean-normalized transcript counts and distance to tissue edge for bins outside the tissue, respectively. Color intensity indicates the number of bins. Source data are provided as a Source Data file.

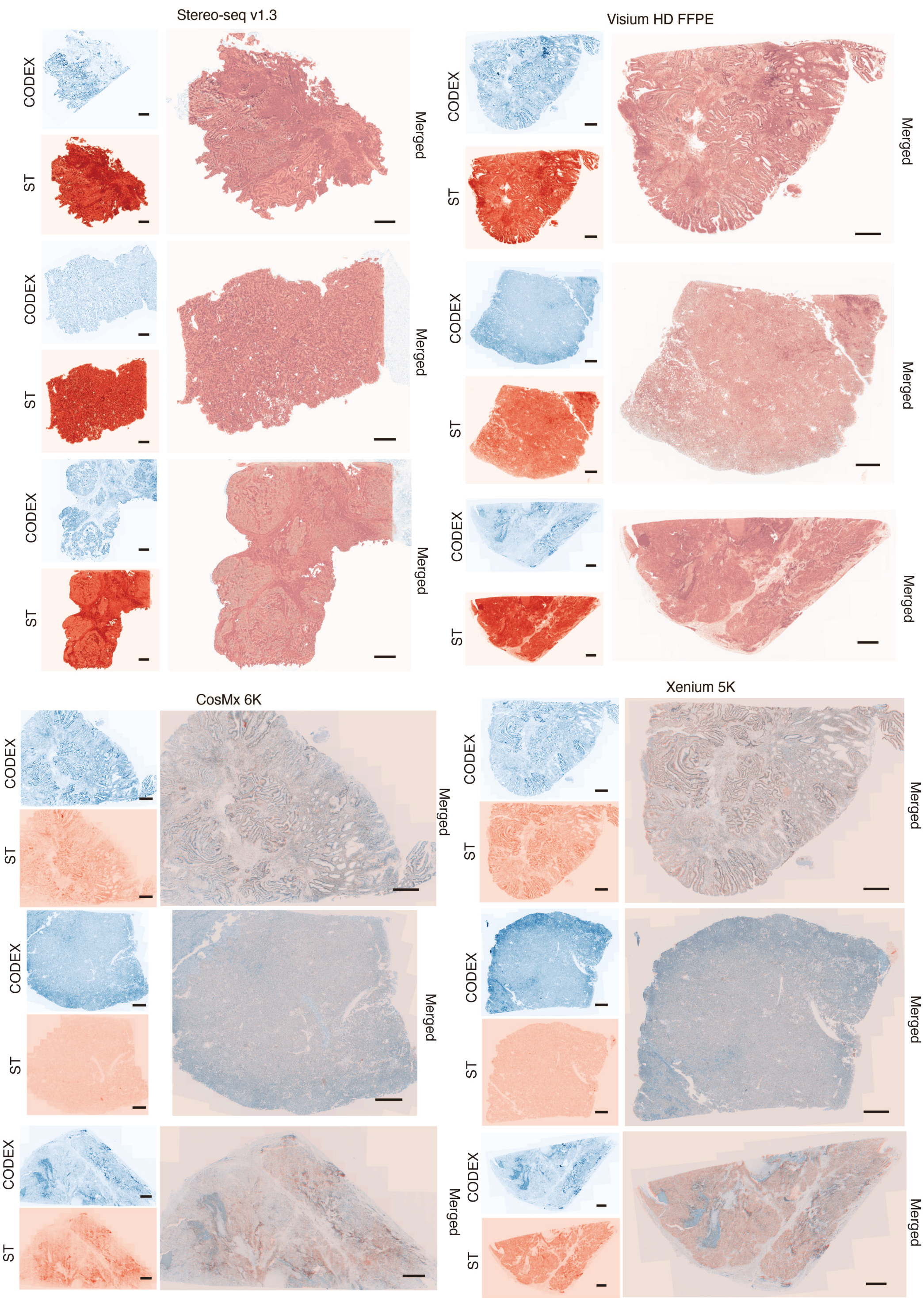

Supplementary Fig. 6 Image alignment between ST and CODEX.
For each group, the top-left panel shows the DAPI channel from CODEX, the bottom-left panel shows the corresponding morphology image from the spatial transcriptomics data, and the right panel shows the merged overlay following image registration. For Stereo-seq v1.3 and Visium HD FFPE, the morphology image corresponds to a grayscale-converted H&E image, whereas for CosMx 6K and Xenium 5K, it derives from the DAPI fluorescence channel. Scale bars, 1 mm.

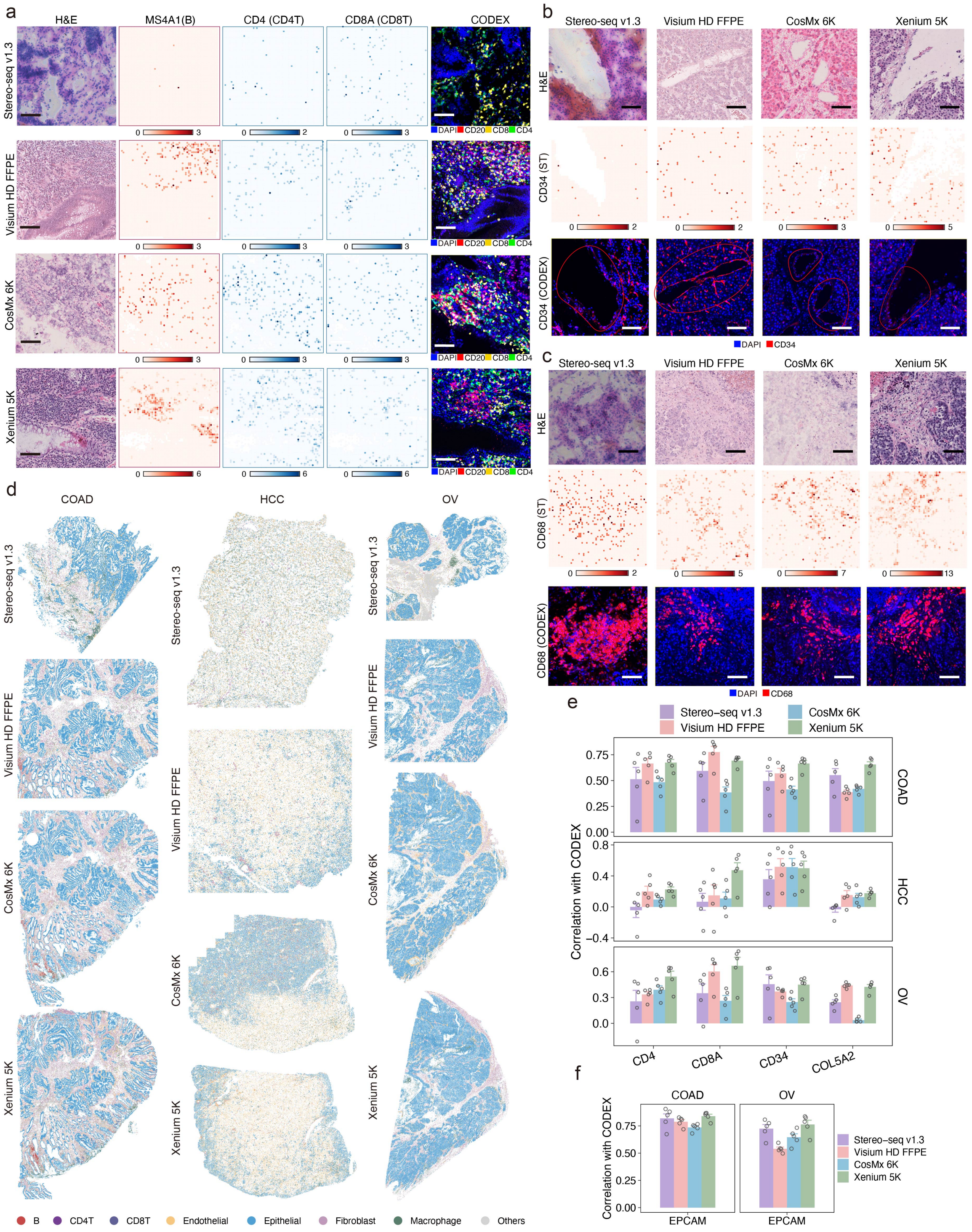

Supplementary Fig. 7 Spatial correlation of transcriptomic and proteomic data across key cell types and tissue structures.

a. Spatial distribution of B cell, CD4^{+} T cell, and CD8^{+} T cell marker genes in selected immune-rich regions (500 × 500 μm) from COAD samples. Adjacent CODEX sections show immunostaining for CD20, CD8, and CD4. H&E images are also shown for reference. Color intensity represents the transcript count in each 8 × 8 μm bin. Scale bars, 100 μm. **b.** H&E staining (top), spatial distribution of CD34 transcripts (middle), and CD34 protein staining (bottom) within ROIs (500 × 500 μm) containing the vascular

structures (highlighted with red solid lines) in HCC. Color intensity represents the transcript count in each 8 × 8 µm bin. Scale bars, 100 µm. **c**. H&E staining (top), spatial distribution of CD68 transcripts (middle), and CD68 protein staining (bottom) within ROIs (500 × 500 µm) containing macrophage aggregates in OV. Color intensity represents the transcript count in each 8 × 8 µm bin. Scale bars, 100 µm. **d**. Spatial distribution of CODEX-annotated cell types. **e-f.** Spatial correlation between CODEX-inferred cell counts and ST-derived marker gene expression for different cell types over the spatial grids. Panel **e** shows the correlations for immune and stromal marker genes, while panel **f** shows the correlations for epithelial marker gene. Pearson correlation coefficients are reported. Hollow circles indicate individual correlation values obtained under different grid sizes (n = 5). Data are presented as mean values +/- SEM. Source data are provided as a Source Data file.

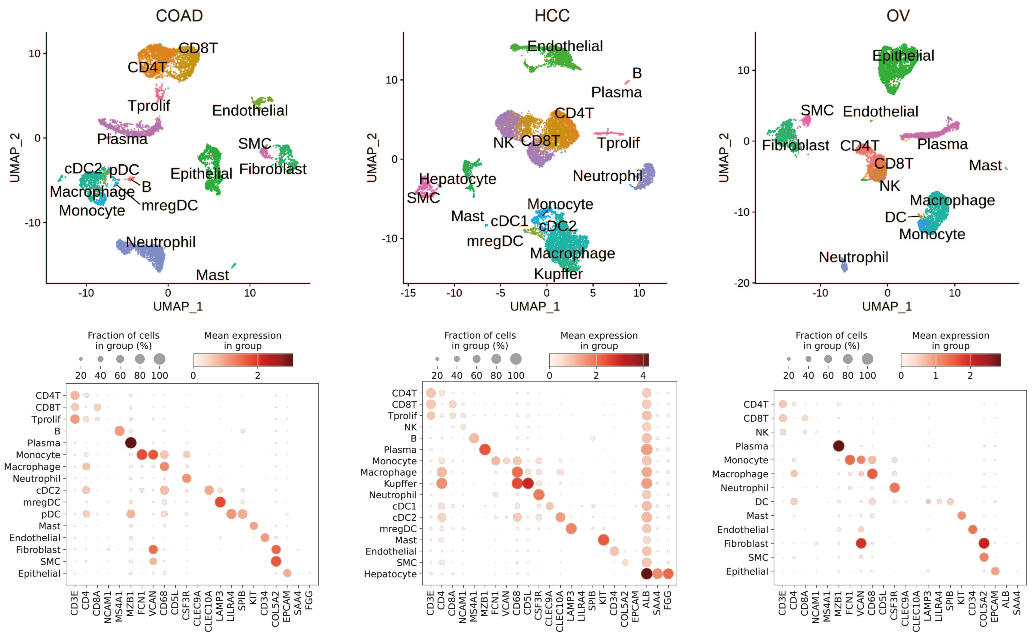

Supplementary Fig. 8 Reference cell type annotations from scRNA-seq. UMAP embeddings of annotated scRNA-seq data for COAD, HCC, and OV, with distinct colors representing major cell types (top). Dot plots (bottom) show the expression of representative marker genes across cell types. Dot size indicates the percentage of cells expressing each gene, and color intensity reflects average expression.

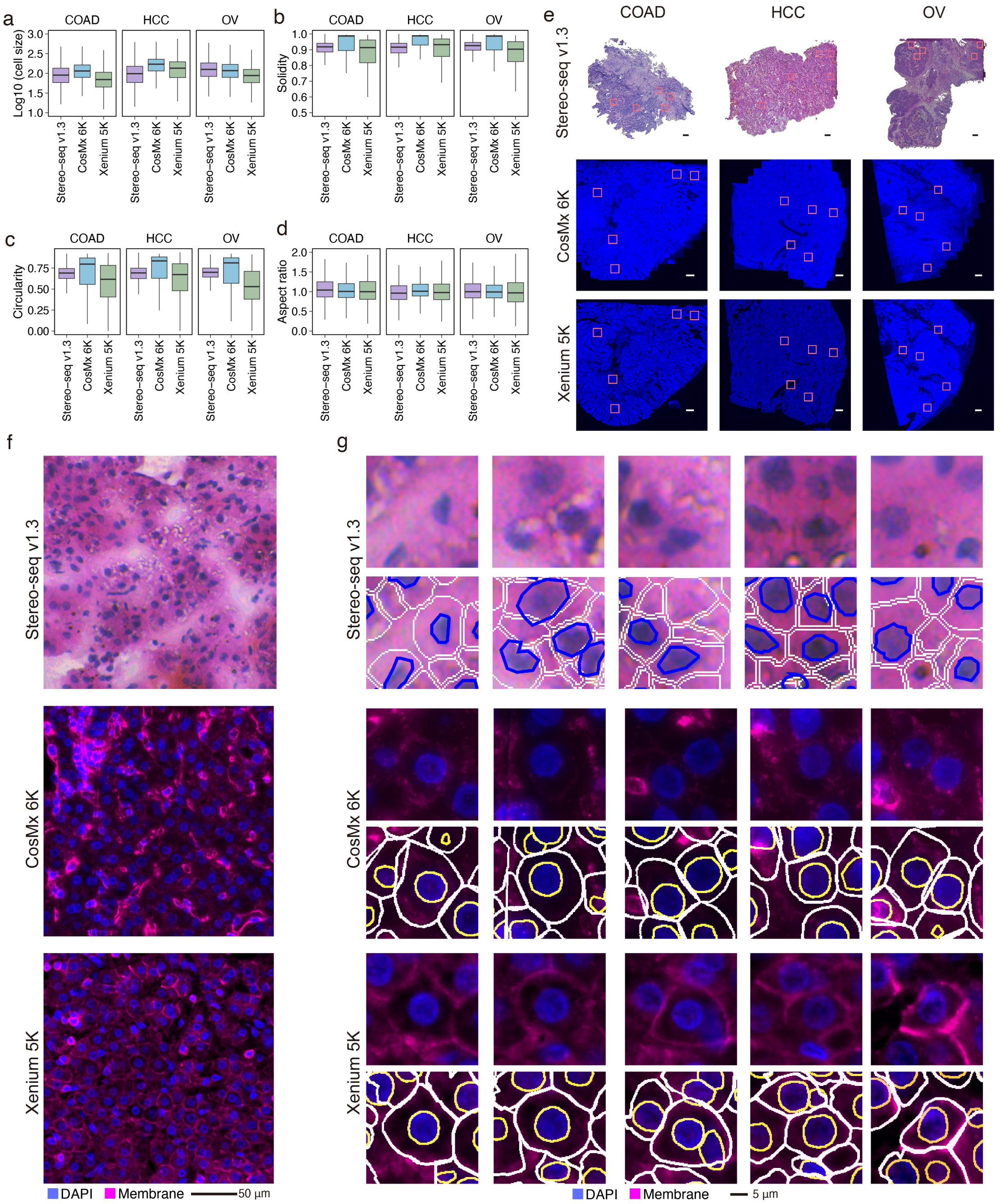

Supplementary Fig. 9 Comparison of cell segmentation.

a-d. Quantitative morphological features of automatically segmented cells, including cell size **(a)**, solidity **(b)**, aspect ratio **(c)**, and circularity **(d)**. Center lines indicate the median value, and lower and upper hinges represent the 25th and 75th percentiles, respectively. The whiskers denote 1.5× the interquartile range. **e.** Spatial distribution of regions (500 × 500 μm) selected for comparing manual nuclear segmentations with automatic cell segmentations across different ST platforms. Scale bars, 500 μm. **f.** Representative regions for comparing cell segmentation across Stereo-seq v1.3 (H&E staining), CosMx 6K and Xenium 5K (multi-channel immunofluorescence staining). Each image represents a 250 μm × 250 μm field of view. **g.** Zoomed-in views of the regions shown in panel (**f**), highlighting segmentation detail. For each platform, the

upper row shows the original images, and the lower row shows overlays of automatic and manual segmentation results. White polygons indicate automatic cell segmentations; blue and yellow polygons indicate manual nuclear segmentations.

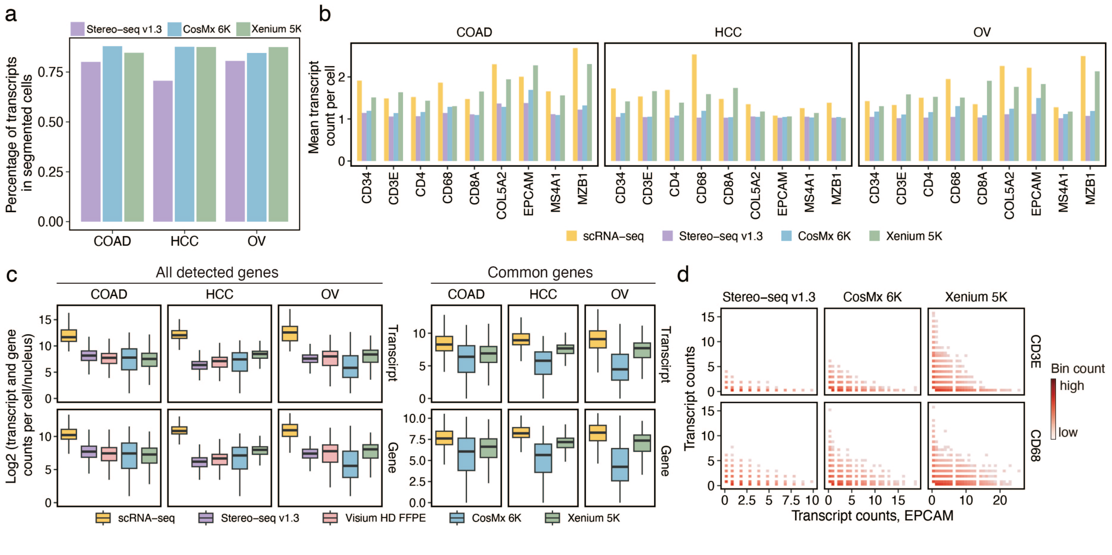

Supplementary Fig. 10 Evaluation of gene detection per segmented cell/nucleus. **a**. Percentage of transcripts localized within segmented cells across platforms. **b**. Mean transcript count per cell for selected marker genes, computed across all cells with non-zero expression values. For ST platforms, the platform-derived automatic segmentations were used. **c**. Log_2-transformed transcript and gene counts are shown for COAD, HCC, and OV samples. For scRNA-seq data, each data point represents a single cell. For ST platforms, each data point corresponds to a nucleus segmented by StarDist. Both all detected genes and common genes shared across platforms were analyzed. Center lines indicate the median value, and lower and upper hinges represent the 25th and 75th percentiles, respectively. The whiskers denote 1.5× the interquartile range. **d**. Joint density plots showing the expression of exclusive marker gene pairs within 8 × 8 µm bins in COAD. Only bins with ≥1 transcript of either marker were included. Color intensity indicates the density of bins. Source data are provided as a Source Data file.

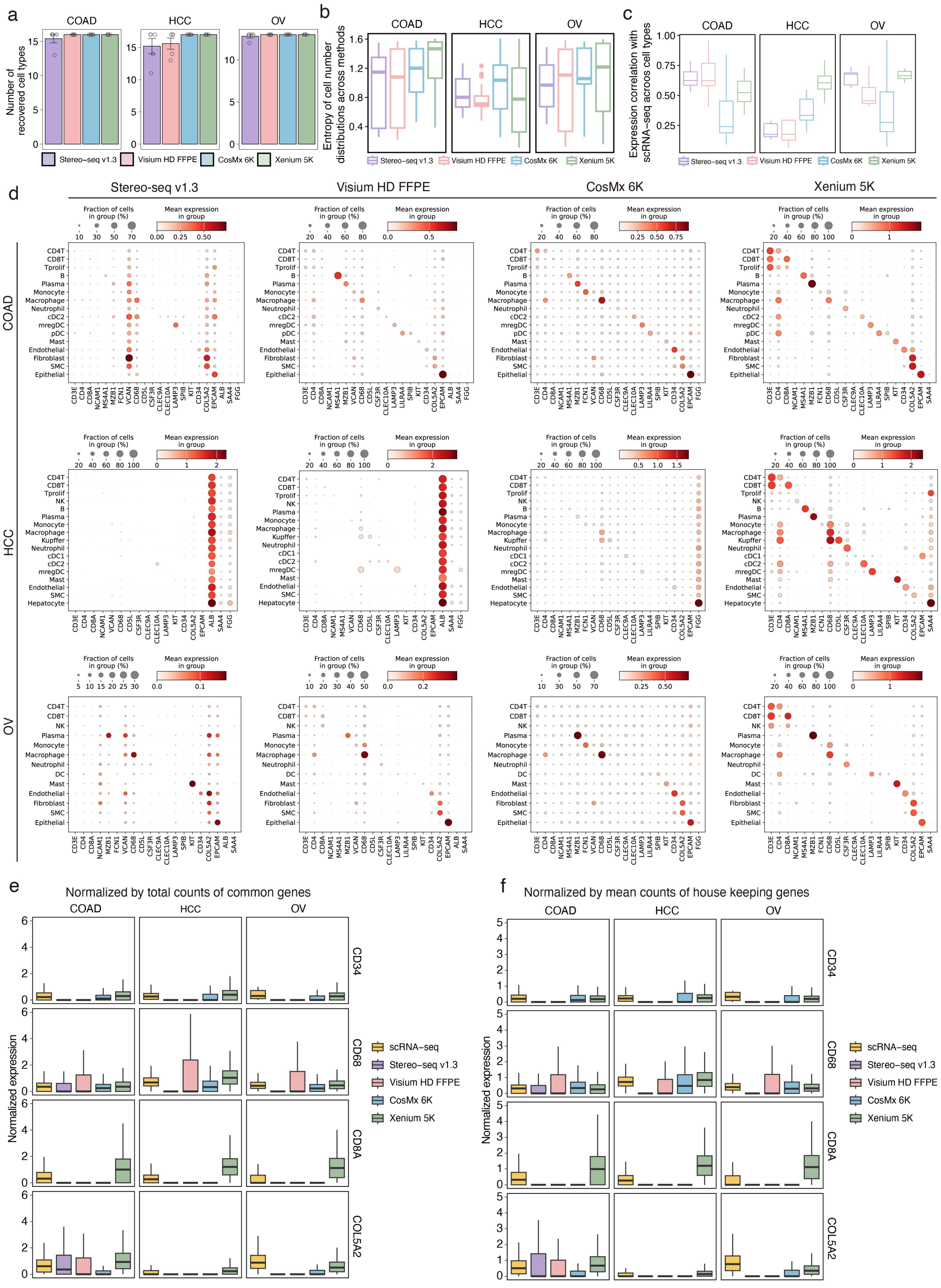

Supplementary Fig. 11 Evaluation of cell type assignment and marker gene expression across platforms.

a. Number of cell types recovered by different ST platforms. Hollow circles indicate cell type count obtained with different annotation tools (n = 5). Data are presented as mean values +/- SEM. **b.** Entropy of cell numbers obtained using various annotation tools for each cell type. Each data point represents one cell type (n = 16 for COAD, n = 17 for HCC, n = 13 for OV). Center lines indicate the median value, and lower and upper hinges represent the 25th and 75th percentiles, respectively. The whiskers denote 1.5× the interquartile range. **c**. Pearson correlation of gene expression between ST data

and scRNA-seq data for each annotated cell type. Each data point represents one cell type (n = 16 for COAD, n = 17 for HCC, n = 13 for OV). Center lines indicate the median value, and lower and upper hinges represent the 25th and 75th percentiles, respectively. The whiskers denote 1.5× the interquartile range. **d**. Expression levels and percentage of lineage marker genes in different cell types. **e**-**f**. Normalized expression of selected marker genes, using either total counts of common genes shared by different platforms (**e**) or mean counts of housekeeping genes (**f**) for normalization. Each boxplot shows the marker expression within its annotated cell type (CD8A for T cells, CD68 for macrophages, CD34 for endothelial cells, COL5A2 for fibroblasts). Center lines indicate the median value, and lower and upper hinges represent the 25th and 75th percentiles, respectively. The whiskers denote 1.5× the interquartile range. Source data are provided as a Source Data file.

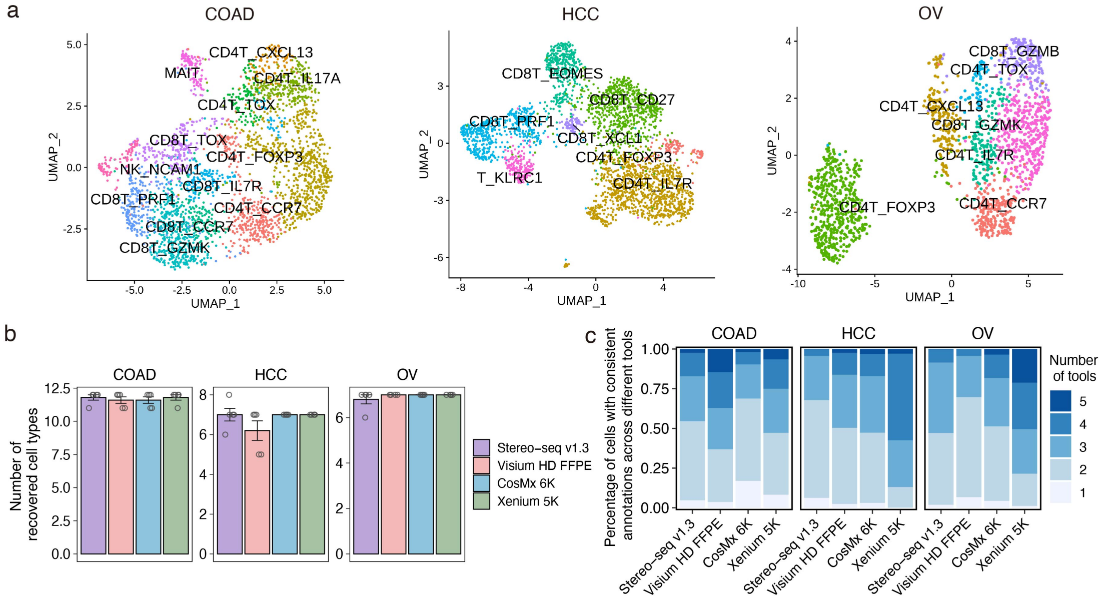

Supplementary Fig. 12 Characterization of T cell subtypes.

a. UMAP representation of scRNA-seq data from three cancer types, colored by T cell subtypes. **b.** Number of T cell subtypes recovered by different ST platforms. Hollow circles indicate cell type count obtained with different annotation tools (n = 5). Data are presented as mean values +/- SEM. **c.** Consistency of automated cell type annotations across five reference-based annotation tools. Bars represent the proportion of T cells annotated as the same subtype by one to five tools. Source data are provided as a Source Data file.

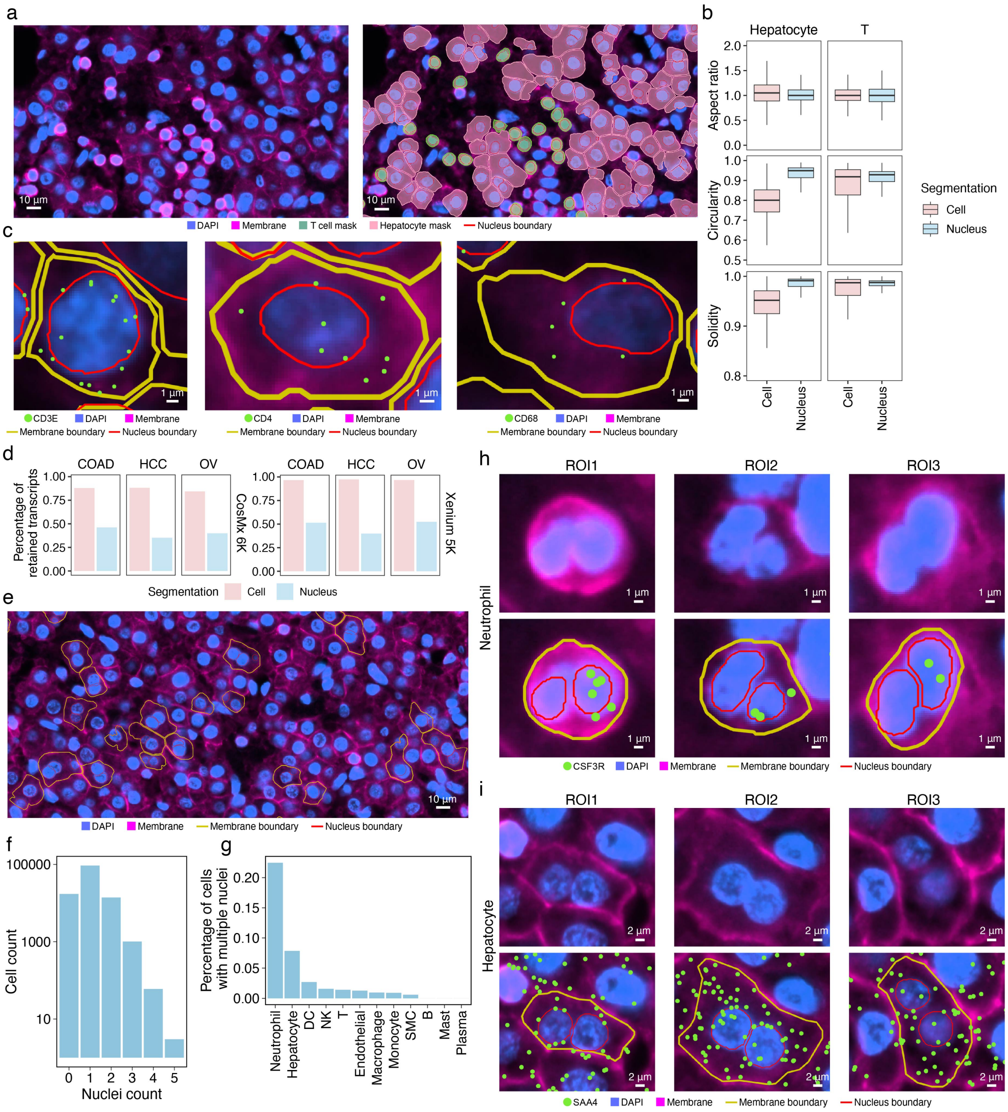

Supplementary Fig. 13 Multimodal cell segmentation improves cell shape delineation and transcript assignment accuracy.

a. Representative field of view from the Xenium 5K HCC sample showing fluorescence image with DAPI and membrane staining (left) and corresponding segmentation results (right), with T cells highlighted in green and hepatocytes in pink. **b**. Comparison of cell shape metrics between cell and nuclear segmentation for hepatocytes and T cells in the Xenium 5K HCC sample. Center lines indicate the median value, and lower and upper hinges represent the 25th and 75th percentiles, respectively. The whiskers denote 1.5× the interquartile range. **c**. Spatial localization of CD3E, CD4, and CD68 transcripts within membrane-based (cell) versus nucleus-based boundaries. **d**. Bar plots showing the proportion of transcripts assigned to either nuclear or cell segmentation masks across cancer types in CosMx 6K and Xenium 5K datasets. Nuclear masks were generated using StarDist. **e**. Representative field of view from the Xenium 5K HCC sample illustrating cells with multiple nuclei. Membrane and nuclear boundaries are outlined. **f**. Histogram showing the distribution of cells by number of nuclei per cell in the Xenium 5K HCC dataset. **g**. Proportion of multinucleated cells across annotated cell types in the Xenium 5K HCC sample. **h-i**. Representative examples of multinucleated neutrophils (**h**) and hepatocytes (**i**) across three ROIs,

showing membrane and nuclear boundaries along with localization of CSF3R (neutrophil marker) and SAA4 (hepatocyte marker) transcripts. Source data are provided as a Source Data file.

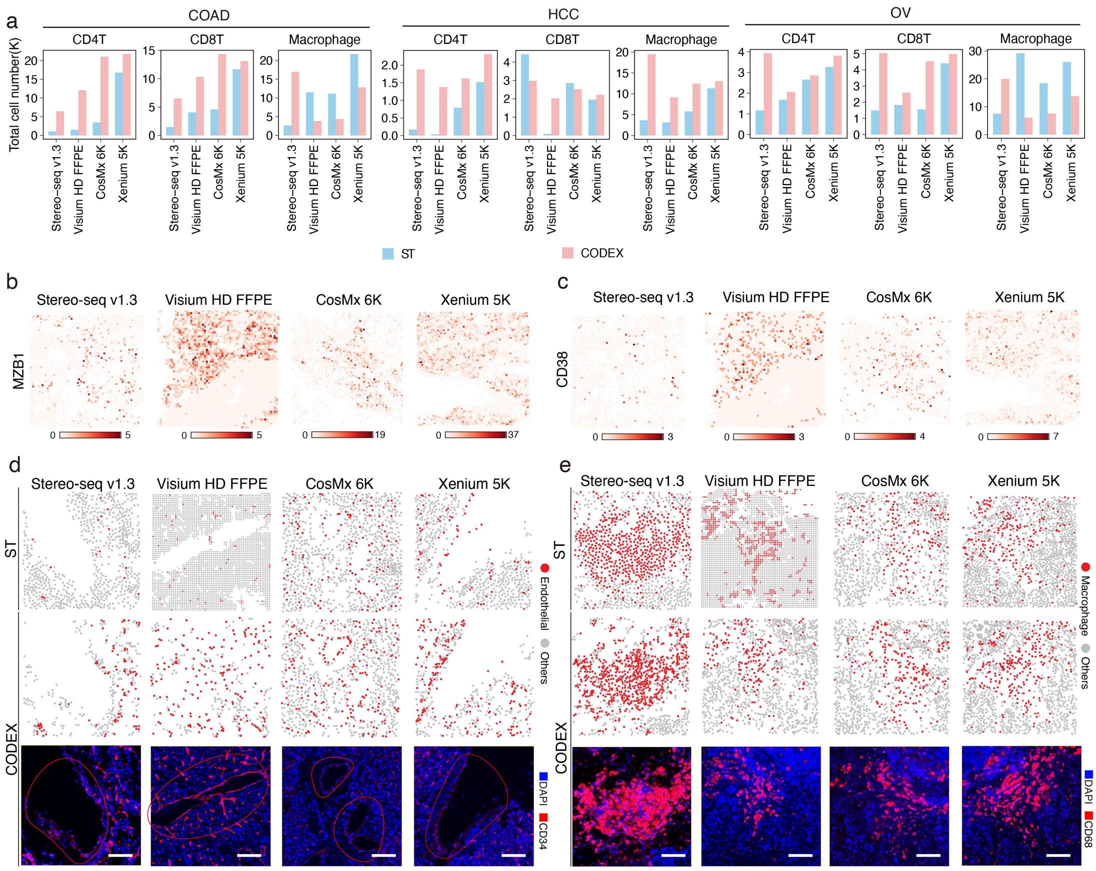

Supplementary Fig. 14 Characterization of spatial distribution for major cell types using ST data and CODEX data.

a. Total number of immune cells detected by different ST platforms and adjacent CODEX across the entire tissue sections. **b-c.** Spatial distribution of MZB1 (**b**), and CD38 (**c**) transcripts within ROIs (500 × 500 μm) showing high lymphocyte infiltration. **d-e.** Spatial distribution of endothelial cells in vascular-rich ROIs (500 × 500 μm) in HCC (**d**) and macrophages in macrophage-aggregated ROIs in OV (**e**). Top: ST-derived cell type annotations; middle: CODEX-derived cell type annotations; bottom: immunostaining of CD34 (**d**) and CD68 (**e**). Scale bars, 100 μm. Source data are provided as a Source Data file.

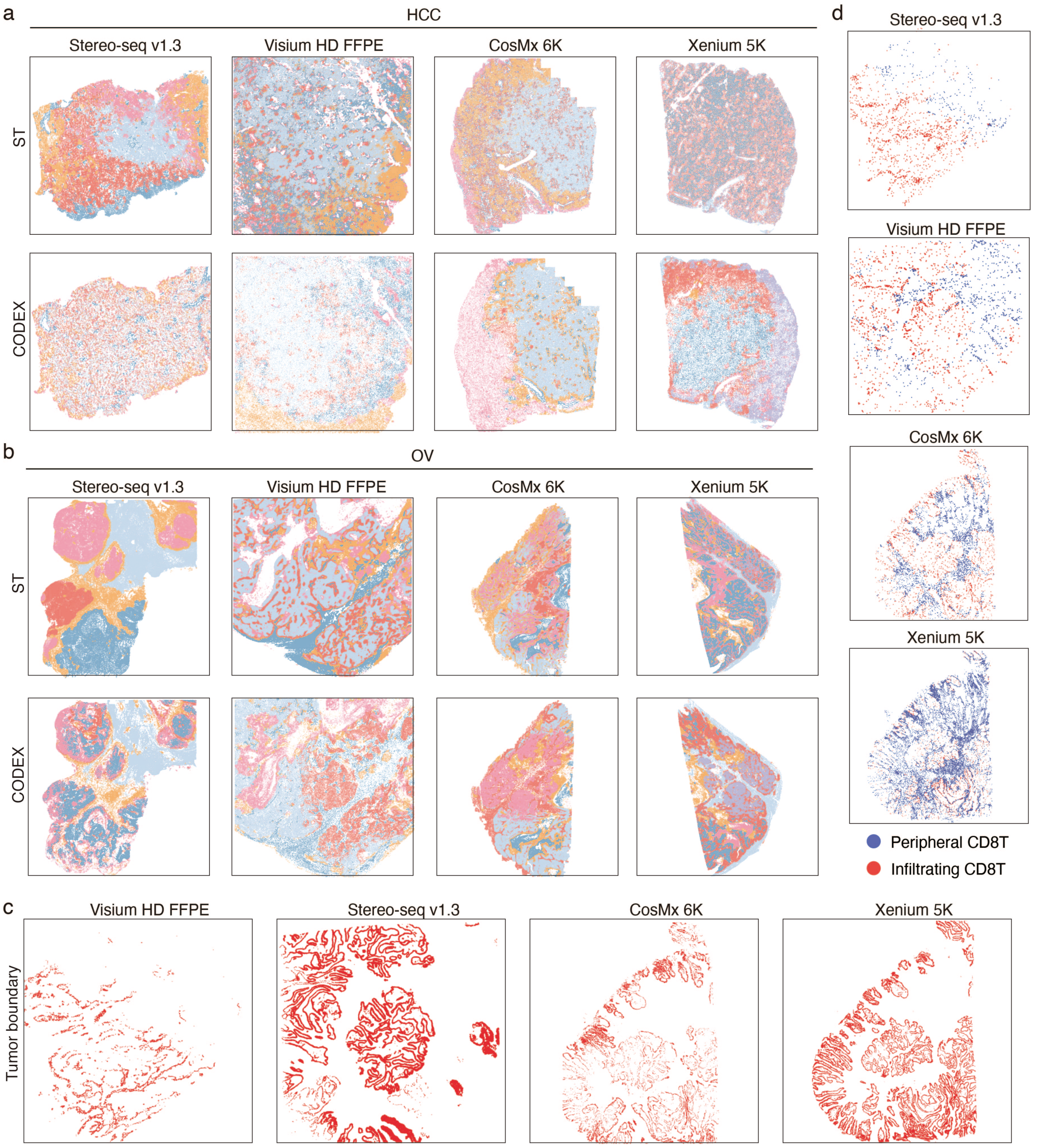

Supplementary Fig. 15 Spatial clustering and distribution of spatially distinct cell subtypes.

a-b. Spatial clustering of ST (top) and CODEX data (bottom) in HCC (**a**) and OV (**b**), with distinct colors representing different spatial clusters. **c.** Spatial distribution of malignant cells localized at the tumor boundary. **d.** Spatial distribution of tumor-infiltrating and peripheral CD8^{+} T cells within the COAD sections.

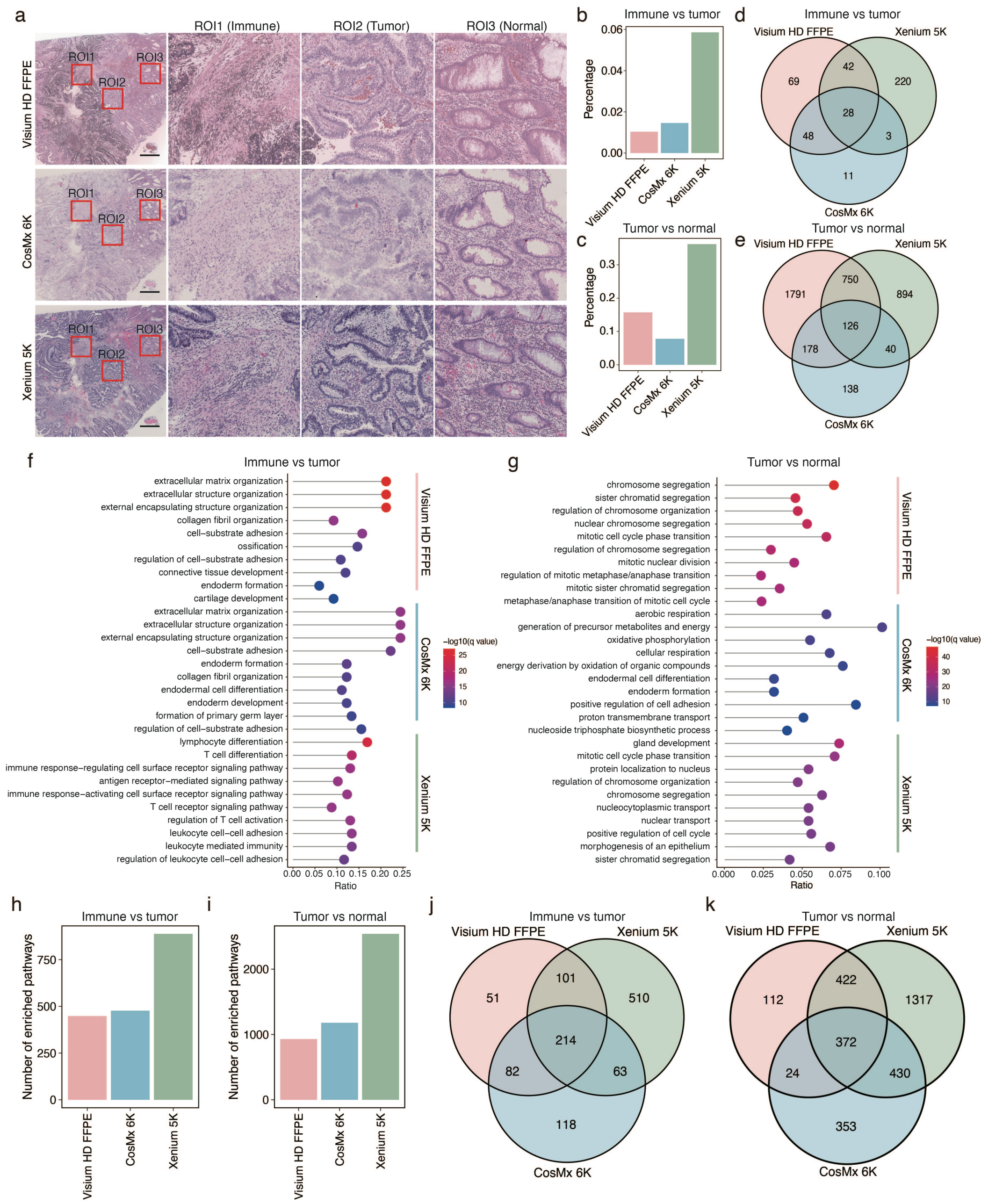

Supplementary Fig. 16 Regional differential gene expression and pathway enrichment analysis.

a. Representative ROIs from the COAD samples across Visium HD FFPE, CosMx 6K, and Xenium 5K platforms. ROI1 corresponds to an immune cell-rich region, ROI2 to a tumor region, and ROI3 to a normal epithelial region. The three columns on the right show magnified views of each ROI (1 mm x 1 mm). Scale bars, 1 mm. **b-c**. Bar chart showing the proportion of DEGs (adjusted p-value ≤ 0.05 and fold change ≥ 2) relative to the total number of genes in each platform's panel, for immune cell-rich region vs. tumor region (**b**) and tumor region vs. normal region (**c**). **d-e.** Venn diagram illustrating the overlap of DEGs identified across the three platforms, for immune cell-rich region vs. tumor region (**d**) and tumor region vs. normal region (**e**). **f-g.** Lollipop plots showing the top 10 enriched GO pathways identified in immune cell-rich regions (**f**) and tumor

regions (**g**). Dot colors represent the -log10 transformed adjusted p-values, and the x-axis indicates the ratio of DEGs involved in each pathway. **h-i**. Bar plots displaying the total number of significantly enriched GO pathways (adjusted p-value ≤ 0.05) detected in immune cell-rich regions (**h**) and tumor regions (**i**) by each platform. **j-k.** Venn diagrams showing the overlap of significantly enriched pathways across platforms in immune cell-rich regions (**j**) and tumor regions (**k**). Source data are provided as a Source Data file.
