# Supplementary material for: Systematic benchmarking of high-throughput subcellular spatial transcriptomics platforms across human tumors
Source: Nat Commun. 2025 Oct 17;16:9232. doi: 10.1038/s41467-025-64292-3 (PMC12534522; doi:10.1038/s41467-025-64292-3)
Supplement: Supplementary file 2 — Reporting Summary [file 41467_2025_64292_MOESM2_ESM.pdf]

nature portfolio

Corresponding author(s): Yuanguang Meng, Zhe Zhang, Zexian Zeng

Last updated by author(s): 2025-6-23

Reporting Summary

Nature Portfolio wishes to improve the reproducibility of the work that we publish. This form provides structure for consistency and transparency in reporting. For further information on Nature Portfolio policies, see our Editorial Policies and the Editorial Policy Checklist.

Statistics

For all statistical analyses, confirm that the following items are present in the figure legend, table legend, main text, or Methods section.

n/a	Confirmed	
☐	☒	The exact sample size (n) for each experimental group/condition, given as a discrete number and unit of measurement
☐	☒	A statement on whether measurements were taken from distinct samples or whether the same sample was measured repeatedly
☐	☒	The statistical test(s) used AND whether they are one- or two-sided<br>*Only common tests should be described solely by name; describe more complex techniques in the Methods section.*
☒	☐	A description of all covariates tested
☒	☐	A description of any assumptions or corrections, such as tests of normality and adjustment for multiple comparisons
☐	☒	A full description of the statistical parameters including central tendency (e.g. means) or other basic estimates (e.g. regression coefficient) AND variation (e.g. standard deviation) or associated estimates of uncertainty (e.g. confidence intervals)
☐	☒	For null hypothesis testing, the test statistic (e.g. F, t, r) with confidence intervals, effect sizes, degrees of freedom and P value noted<br>*Give P values as exact values whenever suitable.*
☒	☐	For Bayesian analysis, information on the choice of priors and Markov chain Monte Carlo settings
☒	☐	For hierarchical and complex designs, identification of the appropriate level for tests and full reporting of outcomes
☐	☒	Estimates of effect sizes (e.g. Cohen's d, Pearson's r), indicating how they were calculated

Our web collection on statistics for biologists contains articles on many of the points above.

Software and code

Policy information about availability of computer code

Data collection	No public data were included in this study.
Data analysis	cellranger (v.7.0.0)<br>spaceranger (v.3.0.0)<br>SAW (v.8.0)<br>OpenCV (v.4.10.0)<br>pysam (v.0.22.1)<br>scikit-learn (v.1.5.2)<br>SimpleITK (v.2.4.0)<br>DoubletFinder (v.2.0.3)<br>Seurat (v.5.1.0)<br>scanpy (v.1.10.3)<br>SELINA (v.0.1)<br>Tangram (v.1.0.4)<br>Celltypist (v.1.6.3)<br>SPACEL (v.1.1.7)<br>TACCO (v.0.4.0.post1)<br>StarDist (v.0.5.0)<br>QuPath (v.0.5.1)

clusterProfiler (v.4.6.2)
Analysis code for this work: https://github.com/zenglab-pku/SPATCH

For manuscripts utilizing custom algorithms or software that are central to the research but not yet described in published literature, software must be made available to editors and reviewers. We strongly encourage code deposition in a community repository (e.g. GitHub). See the Nature Portfolio guidelines for submitting code & software for further information.

Data

Policy information about availability of data

All manuscripts must include a data availability statement. This statement should provide the following information, where applicable:

- Accession codes, unique identifiers, or web links for publicly available datasets
- A description of any restrictions on data availability
- For clinical datasets or third party data, please ensure that the statement adheres to our policy

The raw sequencing data have been deposited in the Genome Sequence Archive at the National Genomics Data Center under accession number HRA011129 (https://ngdc.cncb.ac.cn/gsa-human/browse/HRA011129). The image data have been deposited in BioImage Archive under accession number S-BIAD1900 (https://www.ebi.ac.uk/biostudies/bioimages/studies/S-BIAD1900?query=S-BIAD1900). Both raw and processed data are publicly accessible on the SPATCH website at http://spatch.pku-genomics.org/. Beyond data download, this web server offers tools for data visualization and exploration, enabling users to interactively analyze the datasets.

Research involving human participants, their data, or biological material

Policy information about studies with human participants or human data. See also policy information about sex, gender (identity/presentation), and sexual orientation and race, ethnicity and racism.

Reporting on sex and gender	Samples were collected from two male and one female donors. Sex was determined based on self-reporting. However, no sex- or gender-based analyses were performed in this study, as the sample size was limited and the study was not designed to assess sex-specific differences.
Reporting on race, ethnicity, or other socially relevant groupings	No socially constructed or socially relevant categorization variables (e.g., race, ethnicity, or socioeconomic status) were collected or used in this study. Accordingly, no confounding variables of this type were controlled for in the analysis.
Population characteristics	Samples were obtained from two male and one female adult donors. Diagnostic and treatment information was collected for all participants and is detailed in Supplementary Table 1.
Recruitment	Participants were randomly selected. Samples were collected from the first patients who met the cancer type criteria and consented to participate at the time of the study. Specifically, the inclusion criterion was that the primary tumor type corresponded to colon adenocarcinoma (COAD), hepatocellular carcinoma (HCC), or ovarian cancer (OV), and the tumor was of sufficient size to allow sampling and spatial transcriptomic profiling. No additional selection criteria were applied.
Ethics oversight	This study was approved by the Research and Biomedical Ethical Committee of Peking University (IRB00001052-24061) and conducted following pertinent ethical regulations. All patients provided informed consent for collecting clinical information and tumor samples. All protocols adhered to the Interim Measures for the Administration of Human Genetic Resources, administered by the Ministry of Science and Technology of China. Participants received no compensation for their participation.

Note that full information on the approval of the study protocol must also be provided in the manuscript.

Field-specific reporting

Please select the one below that is the best fit for your research. If you are not sure, read the appropriate sections before making your selection.

[x] Life sciences [ ] Behavioural & social sciences [ ] Ecological, evolutionary & environmental sciences

For a reference copy of the document with all sections, see nature.com/documents/nr-reporting-summary-flat.pdf

Life sciences study design

All studies must disclose on these points even when the disclosure is negative.

Sample size	No formal statistical method was used to predetermine the sample size. The number of samples (n = 3) was determined based on availability of eligible patients and feasibility constraints at the time of study initiation.
Data exclusions	No data were excluded from the analysis.
Replication	No experimental replication was performed in this study due to the limited number of available samples.
Randomization	Participants were not pre-selected. Samples were collected from the first patients who met the cancer type criteria and consented to participate at the time of the study.
Blinding	No group allocation was involved in this study.

Reporting for specific materials, systems and methods

We require information from authors about some types of materials, experimental systems and methods used in many studies. Here, indicate whether each material, system or method listed is relevant to your study. If you are not sure if a list item applies to your research, read the appropriate section before selecting a response.

Materials & experimental systems

n/a	Involved in the study
☐	☒ Antibodies
☒	☐ Eukaryotic cell lines
☒	☐ Palaeontology and archaeology
☒	☐ Animals and other organisms
☒	☐ Clinical data
☒	☐ Dual use research of concern
☒	☐ Plants

Methods

n/a	Involved in the study
☒	☐ ChIP-seq
☒	☐ Flow cytometry
☒	☐ MRI-based neuroimaging

Antibodies

Antibodies used

The following antibodies from the PhenoCycler (formerly CODEX) platform by Akoya Biosciences were used in this study:
PhenoCycler Antibodies, Akoya PN, Clone Name, Lot Number, Dilution factor
Anti-Hu CD20 (AKYP0049, BX007, Alexa Fluor™ 750), 4450018, AKYP0049, B356690, 1 : 200
Anti-Hu CD3e (AKYP0062, BX045, Alexa Fluor™ 647), 4550119, AKYP0062, B330888, 1 : 200
Anti-Hu CD4 (AKYP0048, BX003, Alexa Fluor™ 647), 4550112, AKYP0048, 0923L470, 1 : 200
Anti-Hu CD8 (AKYP0028, BX026, Atto 550), 4250012, AKYP0028, B406228, 1 : 200
Anti-Hu FOXP3 (AKYP0102, BX031, Alexa Fluor™ 647), 4550071, AKYP0102, 0723L235, 1 : 200
Anti-Hu CD56 (AKYP0118, BX028, Atto 550), 4250117, AKYP0118, B398076, 1 : 200
Anti-Hu Pan-Cytokeratin (AKYP0053, BX019, Alexa Fluor™ 750), 4450020, AKYP0053, B330883, 1 : 200
Anti-Hu CD34 (AKYP0088, BX025, Atto 550), 4250057, AKYP0088, 1000000257, 1 : 200
Anti-Hu CD11c (AKYP0051, BX024, Alexa Fluor™ 647), 4550135, AKYP0147, 1122L385, 1 : 200
Anti-Hu CD68 (AKYP0050, BX015, Alexa Fluor™ 647), 4550113, AKYP0050, B363232, 1 : 200
Anti-Hu CD163 (AKYP0114, BX069, Atto 550), 4250110, AKYP0152, 1000000724, 1 : 200
Anti-Hu SMA (AKYP0081, BX013, Alexa Fluor™ 750), 4450049, AKYP0081, B403522, 1 : 200
Anti-Hu MPO (AKYP0113, BX098, Atto 550), 4250083, AKYP0113, 1000000269, 1 : 200
Anti-Hu HLA-A (AKYP0078, BX004, Alexa Fluor™ 750), 4450046, AKYP0078, B397760, 1 : 800
Anti-Hu HLA-DR (AKYP0063, BX033, Alexa Fluor™ 647), 4550118, AKYP0063, B389101, 1 : 200
Anti-Hu IDO1 (AKYP0084, BX027, Alexa Fluor™ 647), 4550123, AKYP0084, 1000000122, 1 : 200

Validation

All antibodies were pre-validated by the manufacturer (Akoya Biosciences) for use in the CODEX multiplexed imaging system. Validation information, including species specificity and application suitability, is available on the manufacturer's website (https://www.akoyabio.com/wp-content/uploads/2021/12/CODEX-screened-antibody-list.pdf).

Plants

Seed stocks

Report on the source of all seed stocks or other plant material used. If applicable, state the seed stock centre and catalogue number. If plant specimens were collected from the field, describe the collection location, date and sampling procedures.

Novel plant genotypes

Describe the methods by which all novel plant genotypes were produced. This includes those generated by transgenic approaches, gene editing, chemical/radiation-based mutagenesis and hybridization. For transgenic lines, describe the transformation method, the number of independent lines analyzed and the generation upon which experiments were performed. For gene-edited lines, describe the editor used, the endogenous sequence targeted for editing, the targeting guide RNA sequence (if applicable) and how the editor was applied.

Authentication

Describe any authentication procedures for each seed stock used or novel genotype generated. Describe any experiments used to assess the effect of a mutation and, where applicable, how potential secondary effects (e.g. second site T-DNA insertions, mosiacism, off-target gene editing) were examined.
