# Supplementary material for: Systematic benchmarking of high-throughput subcellular spatial transcriptomics platforms across human tumors
Source: Nat Commun. 2025 Oct 17;16:9232. doi: 10.1038/s41467-025-64292-3 (PMC12534522; doi:10.1038/s41467-025-64292-3)
Supplement: Supplementary file 3 — Description of Additional Supplementary Files [file 41467_2025_64292_MOESM3_ESM.pdf]

Supplementary Data 1 Metadata of patients
Supplementary Data 2 Timeline for sample collection and processing
Supplementary Data 3 Technical information for all ST platforms used in this study
Supplementary Data 4 Gene sets collected from previous studies
Supplementary Data 5 Intersection between reference gene sets and gene panels of each ST platform
Supplementary Data 6 Marker genes used in the mutually exclusive expression analysis
Supplementary Data 7 Antibodies and cycle information for the CODEX experiment
